# Evaluation of the Biological Efficiency of Silver Nanoparticles Biosynthesized Using *Croton tiglium* L. Seeds Extract against Azoxymethane Induced Colon Cancer in Rats

**DOI:** 10.31557/APJCP.2020.21.5.1369

**Published:** 2020-05

**Authors:** Wael Mahmoud Aboulthana, Noha El-Sayed Ibrahim, Noha Mohamed Osman, Mohamed Mahmoud Seif, Amgad Kamal Hassan, Ahmed Mahmoud Youssef, Amal Mostafa El-Feky, A A Madboli

**Affiliations:** 1 *Biochemistry Department, Genetic Engineering and Biotechnology Division, National Research Centre, Dokki, Giza, Egypt. *; 2 *Microbial Biotechnology Department, Genetic Engineering and Biotechnology Division, National Research Centre, Dokki, Giza, Egypt. *; 3 *Cell Biology Department, Genetic Engineering and Biotechnology Division, National Research Centre, Dokki, Giza, Egypt. *; 4 *Toxicology and Food contaminants, Food Industry and Nutrition Division, National Research Center, Dokki, Giza, Egypt. *; 5 *Packaging Materials Department, National Research Center, Dokki, Giza, Egypt. *; 6 *Pharmacognosy Department, Pharmaceutical and Drug Industries Research Division, National Research Centre, Dokki, Giza, Egypt. *; 7 *Animal Reproduction and Artificial Insemination Department, Veterinary Division, National Research Centre, Dokki, Giza, Egypt . *

**Keywords:** Colorectal Cancer, Croton tiglium Seeds, Green Nanotechnology, Electrophoresis, Isoenzymes

## Abstract

**Background:**

Colorectal cancer (CRC) is considered as the most common type of gastrointestinal cancers. Chemotherapy became limited due to the adverse side effects. Therefore, the most effective *Croton tiglium* extract was selected to be incorporated by silver nanoparticles (Ag-NPs) then evaluated against colon cancer induced by azoxymethane (AOM) in rats.

**Methods:**

Different hematological and biochemical measurements were quantified in addition to markers of oxidative stress. Specific tumor and inflammatory markers were assayed. Colonic tissues were examined histopathologically in addition to immunohistochemistry (IHC). Native proteins and isoenzymes patterns were electrophoretically assayed beside expression of Tumor Protein *P*^53^ (*TP*^53^) and *Adenomatous Polyposis Coli (APC)* genes in colonic tissues.

**Results:**

It was found that AOM caused significant (P≤0.05) elevation in the hematological and biochemical measurements. *C. tiglium* nano-extract restored these measurements to normalcy. Tumor and inflammatory markers elevated significantly (P≤0.05) in sera of AOM induced colon cancer group in addition to increasing peroxidation products with decline in antioxidant enzymes activities in colon tissues. Nano-extract restored these measurements to normalcy in post-treated group. Histopathological study revealed that nano-extract minimized severity of inflammatory reactions in all nano-extract treated groups and prevented anti-Keratin 20 antibody expression in post-treated group. The lowest similarity index (SI%) values were noticed with electrophoretic protein (SI=71.43%), lipid (SI=0.00%) and calcium (SI=75.00%) moieties of protein patterns, catalase (SI=85.71%), peroxidase (SI=85.71%), α-esterase (SI=50.00%) and β-esterase (SI=50.00%) isoenzymes in colon cancer group. Furthermore, AOM altered the relative quantities of total native bands. The nano-extract prevented the alterations that occurred qualitatively in nano-extract post-treated group and quantitatively in all nano-extract treated groups. Levels of *TP*^53^ and *APC* gene expression increased in AOM injected group and nano-extract restored their levels to normalcy in the post-treated group.

**Conclusion:**

*C. tiglium* nano-extract exhibited ameliorative effect against the biochemical and molecular alterations induced by AOM in nano-extract post-treated group.

## Introduction

Colorectal cancer (CRC) is categorized as one of the most common types of gastrointestinal cancers that studied extensively in the world. It is considered as the third highest cause of cancer death (Shelton, 2002). The CRC pathogenesis involves initiation of epithelial cells to a cancerous state with defined precancerous intermediaries by sequential and multistep progression (Raju, 2008). Aberrant crypt foci are abnormal clusters identified early in the lining of colon and rectum before colorectal polyps as precancerous lesions during colon carcinogenesis (Kim et al., 2008).

Success of cancer chemotherapy is limited due to the adverse effects induced by the drug, multidrug resistance and off-targets binding (Moorthi et al., 2011). In order to develop new cancer chemotherapy, it is necessary to induce cancer tumors in rats to evaluate the efficacy of the potential anti-cancer drugs. Many carcinogens are able to induce colon tumors in rats but azoxymethane (AOM) has been most widely used (Chen and Huang, 2009). Incidence of colon tumors induced by two or three subcutaneous injections of AOM takes 7-9 months to induce sufficient tumors (Femia and Caderni, 2008). When an inflammatory agent is administered via drinking water to the AOM-treated rats, it accelerates the induction process and it was postulated that dextran sodium sulfate (DSS) is the most suitable inflammatory agent which is able to develop number of colon tumors within a short time period (Gupta et al., 2007).

Biomarkers such as carcinoembryonic antigen (CEA) and carbohydrate antigen (CA) 19-9 have the characteristics that enable them to detect the progression of CRC and gastrointestinal tumor at earlier stages (Duffy, 2001 ; Tanaka et al., 2010). Alterations of specific oncogenes and tumor suppressor genes play a role at different stages of carcinogenesis process (Luceri et al., 2000). Protein encoded by adenomatous *polyposis coli (APC)* gene participates in signaling transduction pathway. Mutations in the* APC* gene play a rate-limiting role in majority of sporadic colorectal cancers and hypermethylation of its promoter is closely related to cancer development (Pan et al., 2009). A tumor suppressor protein encoded by tumor suppressor *P*^53^* (TP*^53^*)* gene that regulates expression of the genes involving apoptosis, angiogenesis and genome maintenance. Mutations in *TP53 *genes appear to occur at relatively late stage of colorectal cancer (Mills, 2005).

The synthetic antioxidants have restrictions for medicinal uses, as they are suspected to be carcinogenic (ElFar and Taie, 2009). Recent studies in medicinal plants field aimed to find suitable natural antioxidants for therapeutic purposes. These antioxidants can reduce free radicals induced by tissue injury (Erdemoglu et al., 2006). *Croton tiglium* L is one of the largest genera of flowering plants which are widely used in ethnomedicine for the treatment of several cancer diseases (Nath et al., 2013). It exhibited high antioxidant activity due to the presence of some other phytochemicals such as ascorbic acid, tocoferol and other pigments (Sengul et al., 2009). The croton oil isolated from *C. tiglium* seeds used for treatment of the solid tumors due to presence of 12-O-tetradecanoylphorbol-13-acetate (the major active constituent) that is able to stimulate apoptosis in prostate, breast, colon and lung cancers (Rickard et al., 1999).

It was emphasized that aqueous *C. tiglium* extract can cause increase in plasmid DNA strand breaking. This increase depends on the concentration used of the extract. Therefore, the plant extract needs to be tested to determine the required dose before directed to therapeutic purposes (Yumnamcha et al., 2014). Recent study showed that* C. tiglium *seeds extracts have no significant alterations in the biochemical measurements such as total protein, albumin and globulins of rats fed with *C. tiglium *seeds extracts. However, these extracts have significant effect on some haematological indices (El-Kamali et al., 2015).

Most of the biologically active components absorbed slowly due to their high molecular weights which decreases their ability to cross the cellular membrane and hence decreases their efficacy and bioavailability. Therefore, integration of the traditional medicinal plants with nanotechnology can overcome such problems by increase their bioavailability and reduce their toxicity (Bonifácio et al., 2014; Mamillapalli et al., 2016). Development of nano-extracts containing metal nanoparticles (M-NPs) showed great promise to solve their inherent stability problem. Incorporation of M-NPs into polymeric matrices showed valuable properties in many practical applications (Rozenberg and Tenne, 2008). The plant extracts with M-NPs increased the total phenolic compounds and exhibited good antioxidant activity at lower concentrations (Johnson et al., 2014 ; Shousha et al., 2019). Therefore, it showed a higher antioxidant and antimicrobial activity as compared to plant extract solely (Abdel-Aziz et al., 2014). 

From this point of view, the study was designed to develop novel strategies for prevention of colon cancer by mean of green nanotechnology through incorporating Ag-NPs into *C. tiglium* extract to enhance its antioxidant efficiency as suggested by Aboulthana et al., (2019) then to evaluate nano-extract against colon cancer induced by AOM in rats.

## Materials and Methods

*Silver C. tiglium nano-extract administration*

The aqueous silver *C. tiglium* nano-extract was prepared and characterized by advanced confirmatory technique and administrated orally by stomach tube at a dose of 6.5 ml/Kg body weight (b.wt) (1/20 of Median Lethal Dose (LD_50_) (Aboulthana et al., 2019).

*Induction of colon cancer in rats*

Rats were injected by a single injection of AOM at a dose of 20 mg/kg b.wt interperitoneally (i.p.) to induce colon carcinogenesis chemically then rats were given 2% DSS in drinking water for 7 days after one week of AOM injection. Histological examination for injected rats was done from 8th week of AOM injection to detect disease induction (Yoshimi et al., 2009). It was found that the disease induced after 12 weeks. The induced rats were consequently used to evaluate efficacy of the prepared silver *C. tiglium* nano-extract against colon cancer. 

*Experimental design*

Healthy thirty-six adult male Wistar rats (weighting 120 - 150 g) were housed in cages (six per cage). Rats were provided with water ad libitum and maintained under normal nutritional and environmental conditions at 25 ± 2°C. The experimental procedures were carried out according to the ethical protocol and guidelines approved by the institutional animal care of National Research Centre, Dokki, Giza, Egypt. 

Rats were randomly divided into Control group: Rats were fed with normal diet and received distilled water for 21 days, Silver *C. tiglium* nano-extract treated group: Rats were fed with normal diet associated with the treatment with *C. tiglium* nano-extract orally at a dose of 6.5 ml/Kg b.wt for 21 days, Colon cancer induced group: Rats were injected with AOM at a dose of 20 mg/kg i.p., Silver *C. tiglium* nano-extract pre-treated group: Rats were treated with *C. tiglium* nano-extract orally at a dose of 6.5 ml/Kg b.wt for 21 days followed by injecting AOM i.p. for another 12 weeks, Silver *C. tiglium* nano-extract simult.-treated group: Rats treated simultaneously with AOM i.p. and treated orally by *C. tiglium* nano-extract and Silver *C. tiglium* nano-extract post-treated group: Rats were injected AOM i.p. for 12 weeks followed by the treatment with *C. tiglium* nano-extract orally at a dose of 6.5 ml/Kg b.wt for another 21 days.

*Collection of blood samples and tissues*

Rats were anaesthetized and sacrificed by decapitation at end of the experimental period then heparinized blood samples were collected from retro orbital plexus from each rat for the hematological measurements and serum samples were collected for biochemical measurements by centrifuging blood samples for 15 min at 4,000 rpm. In addition, colon tissues were collected then excised and washed in ice-cold saline. All collected colon tissues were autopased and splitted into 2 portions. Portion (I) was immediately fixed in 10% formal saline until the histopathological examination. Portion (II) was immediately washed in physiological saline then homogenized in Tris-HCl buffer (0.01 M and pH 7.4). Aliquots of the clear supernatants were used for measuring the inflammatory and oxidative stress markers and for undergoing the different electrophoretic patterns.

*Hematological and biochemical measurements*

Heparinized blood samples were analyzed on an automatic blood analyzer (ABX Micros 60 manufactured by HORIBA ABX SAS) to quantify hemoglobin (HB), red blood cells (RBCs), hematocrit (HCT), corpuscular volume (MCV), mean corpuscular haemoglobin (MCH), mean corpuscular haemoglobin concentration (MCHC), red blood cell distribution width (RDW), mean platelet volume (MPV) and platelet count (PLT), white blood cells (WBCs) and differential blood cells (lymphocytes, monocytes and granulocytes). While serum blood samples were used to estimate all traditional biochemical parameters (liver enzymes (alanine transaminase (ALT) and aspartate transaminase (AST), lipid profile (total cholesterol (TC) and triglycerides (T.Gs)), renal functions (urea and creatinin) and protein profile (total protein and albumin)) spectrophotometrically by colorimetric methods following instruction of the commercial kits purchased from Spectrum Diagnostics Egyptian Company for Biotechnology (Cairo, Egypt).

*Tumor markers*

Tumor markers specific for colon cancer including Carcinoembryonic Antigen (CEA) and Carbohydrate Antigen (CA) 19-9 were assayed quantitatively in sera of all groups using Enzyme Linked Immune Sorbent Assay (ELISA) technique.

*Inflammatory markers*

Markers of the inflammatory response specific for colon cancer were quantified through measuring C-reactive protein (C-RP) level and Myeloperoxidase (MPO) activity. C-RP level was determined in serum samples and expressed as mg/L using commercial enzymatic kit (Bio-Diagnostic, Cairo, Egypt). While, MPO activity was determined in supernatant of colon tissue homogenate and expressed by Δ/min/mg protein (Correia et al., 2012).

*The major cellular contents in colon tissues homogenates*

Three cellular contents were assayed as following calcium concentration was determined by using fully automated with microprocessor-controlled electrolyte system (Sensa Core’s ST-200 CL) and Lactate dehydrogenase (LDH) activity was done according to Babson and Babson (1973). Finally, Glucose level was measured by enzymatic colorimetric method following instructions of the Kit manufacture (Bio-Diagnostic, Cairo, Egypt).

*Markers of Oxidative Stress*

All these markers were assayed by using clear supernatants of colon tissue homogenates. These assays including: i) Products of the peroxidation reactions: Lipid peroxidation product (LPO) was determined and expressed as malondialdehyde (MDA) which is the end product of peroxidation reaction and the result was expressed as (nmol/g wet tissue) (Ohkawa et al., 1979). While total protein carbonyl content (TPC) was quantified spectrophotometrically and expressed as nmol of reactive carbonyl compounds per mg protein of tissue (Levine et al., 1994). ii) Antioxidant enzymes: Total Antioxidant Capacity (TAC) was determined and expressed as mM/L (Koracevic et al., 2001). While Catalase Activity (CAT) was assayed and expressed as Unit per gram of tissue (Aebi, 1984). Finally, Glutathione Peroxidase activity (GPx) was measured and expressed as Unit per gram of tissue (Paglia and Valentine, 1967). 

*Statistical Analysis*

All data were statistically analyzed by one-way analysis of variance test (one-way ANOVA) using the Statistical Package for Social Sciences (SPSS for windows, version 11.0) followed by Bonferoni test. Results were expressed as mean ± standard error (SE). The differences between the groups were considered statistically significant when a “*P*” value of less than 0.05.

*Histopathological Examination*

After sacrifice, specimens of colonic tissues were autopsied from different groups then studied histopathologically (Banchroft et al., 1996). All samples were immediately fixed in 10% formal saline for 24 hr and washed in tap water then dehydrated in serial dilutions of alcohol solutions. Tissue fragments were then cleared in xylene and embedded in the paraffin bees wax blocks that were prepared for sectioning at 4 microns thickness by slidge microtome. The tissue sections were collected on glass slides and deparaffinized then stained by hematoxylin and eosin (H and E) stain for the examination under light electric microscope to confirm occurrence of the induction process. The histopathological changes were scored (Dommels et al., 2007). A rating score between 0 (no damage) and +++ (maximal damage) was assigned for carefully investigated section. Also, the induction process was confirmed by the immunohistochemistry (IHC) technique for detection and localization of the anti-Keratin 20 antibody in colon tissue (Haines and Clark, 1991).

*Electrophoretic Assays*

Known weights of colon tissues (0.2 gm) were freezed rapidly in liquid nitrogen then homogenized in 1 ml water-soluble extraction buffer. The homogenates were centrifuged at 10.000 rpm for 5 min. and the clear supernatants transferred into another tubes. Known volumes of the individual homogenates supernatants were pooled together and used as one sample for each group. Concentration of total protein was determined in all pooled samples (Bradford, 1976). Protein concentration must be equal in all wells during all electrophoretic assays. These assays including:

i) Electrophoretic protein pattern: Native proteins were separated electrophoretically through Polyacrylamide Gel Electrophoresis (PAGE) (Laemmli, 1970 ; Darwesh et al., 2015).

ii) Electrophoretic lipid moiety of native proteinpattern: Native gels were stained for detecting lipid moiety of native protein with Sudan Black B (SBB).The stained lipoprotein bands appeared as black bands. The Rf, B% and Qty% of lipoprotein bands were determined Subramaniam and Chaubal (1990).

iii) Electrophoretic calcium moiety of native protein pattern: Native gels were stained for detecting calcium moiety of native protein using alizarin Red ‘S’. The stained calcium moieties appeared as yellow bands. The Rf, B% and Qty% of the calcium moiety bands were determined (Zacharia and Kakati, 2004 ; Abd Elhalim et al., 2017 ; Abulyazid et al., 2017).

iv) Electrophoretic isoenzymes (zymography): Non-denatured PAGE is used for detecting the enzyme activity through identification of the enzyme subunits. Firstly, electrophoretic catalase pattern (CAT) and Electrophoretic peroxidase (POX) pattern were assayed for native gel after electrophoretic run by incubating with H_2_O_2_ as substrate then stained.The stained CAT subunits appeared as yellow bands in the gel (Gregory and Fridovich, 1974 ; Siciliano and Shaw, 1976). While, the stained POX subunits appeared as brown bands in the gel (Rescigno et al., 1997). Finally, Electrophoretic esterase (EST) pattern was carried out to localize in-gel EST activity by incubating gel in conditioning buffer then staining in reaction mixture consisting of α, β-naphthyl acetate (5.58 X 10-3 mM, pH 7.5) as substrates along with dye coupler Fast Blue RR. The α-naphthyl acetate used as substrate for α-esterases which appeared as brown bands and β-naphthyl acetate used as substrate for β-esterases which appeared as dark pink bands Ahmad et al., (2012).

*Data Analysis*

PAGE plates were photographed and analyzed using Quantity One software (Version 4.6.2). The relative mobility (Rf), band percent (B%) and relative band quantity (Qty%) of the electrophoretically separated bands were determined in addition to the molecular weights (Mwts) which were determined by this program in comparison to marker of standard molecular weights with regularly spaced bands ranging from 6.458 to 195.755 KDa. The similarity index (SI%) and genetic distance (GD%) were calculated (Nei and Li, 1979). 

*Molecular Assay*

*RNA extraction and cDNA synthesis*

Total RNA was extracted from colon tissues using easy-spinTM Total RNA Extraction Kit (iNTRON Biotechnology). The RNA concentration and purity were determined using NanoDrop^®^ (ND-1000 Spectrophotometer, NanoDrop Technologies Inc, Delaware, USA). Purity of the RNA yield was accepted in all samples (A260/A280 absorbance ratio of between 1.85 and 2.0). All RNA samples were normalized to1,000 ng/μL, DNase treated (DNaseI, Thermoscientific) and reverse transcribed into First Strand cDNA using Power cDNA Synthesis Kit (iNTRON Biotechnology) according to manufacturers’ protocols.

*Primer design*

Specific primers for *APC*

(F: 5’-TGGAGAGAGAACGAGGTATT-3’; B: 5’-CTTCCATCACTTTGGCTATCT-3’) and

*TP* ^53^

(F: 5’- ACTTTAGGGCTTGTTATGAGAG-3’; B: 5’-CAGGAACCAGTTTGCATAGA-3’) genes were designed on the bases of the Rattus norvegicus sequence Gene bank accessions NM_012499 and NM_030989 respectively using Primer Quest Tool and synthesized by Macrogen company. The designed primers were used for Real time quantitative PCR (qRT-PCR). For gene expression analysis, Beta actin (Actb) was used as endogenous control and its primers (F: 5’-TGTGGATTGGTGGCTCTATC-3’; B: 5’-CAGTAACAGTCCGCCTAGAA-3’) were designed based on sequence of Gene bank NM_031144.

*Gene expression using qRT-PCR*

Expression of the *APC* and *TP*^53^ genes were determined in colon tissues of control and different treated groups. A master mix was prepared for each assay containing 10μL of 2X SYBR green PCR mix (TOPreal™ qPCR 2X PreMIX, enzynomics), 1 ul of 10uM primer sets and nuclease-free water. For each sample, a 25 μL reaction volume of mastermix containing 1 ul of cDNA was prepared. The qPCR was performed by Qiagen’s real-time PCR system (Rotor-Gene Q) using default cycling conditions : 40 cycles of an initial activation step of 95°C for 15 min followed by 95°C for 10 s (melt), 57°C for 15s (anneal) and 72°C for 30s (extend). The mean Cycle threshold (Ct) values were calculated and then normalized to Actb using ΔCt method. Changes in relative expression were calculated using the 2^-ΔΔCt ^method [K.J.Livak ,T.D. Schmittgen , methods 25 (4) (2001) 402-408].

*Statistical analysis*

Molecular data were statistically analyzed using the SPSS computer program (SPSS Inc., Chicago, Illinois, USA). Levels of the relative gene expression were compared between control and other treated groups using the non-parametric Mann-Whitney test. Results were reported as the arithmetic mean ± SE, and P value less than 0.05 is considered statistically significant (***=P<0.001, **=P<0.01, *=P<0.05 and ns=statistically non- significant).

## Results

Although the present study was designed to reveal efficiency of *C. tiglium* extract after incorporating Ag-NPs against colon cancer induced by AOM, it was found that AOM caused adverse effects at the hematological and biochemical levels during the induction process.

*Hematological Measurements*

As shown in Table 1, AOM caused no significant alterations in the hematological measurements related to indices of red blood cells (RBCs, HB, HCT, MCV, MCH and MCHC). While it caused a significant (P≤0.05) elevation in RDW, MPV, PLT and WBCs with its differential count levels when compared to control group. Although treatment with *C. tiglium* nano-extract caused significant (P≤0.05) decrease in levels of all these measurements in all nano-extract treated groups as compared to AOM induced colon cancer group, it restored these measurements to normal values in post-treated group.

*Biochemical Measurements*

As reported in Table 2, it was found that activities of liver enzymes (ALT, AST and ALP) in addition to TC and T.Gs levels increased significantly (P≤0.05) in sera of AOM induced colon cancer group when compared to control group. While, these biochemical measurements levels showed significant (P≤0.05) decrease in silver *C. tiglium* nano-extract treated groups as compared to AOM induced colon cancer group. Furthermore, AOM affected kidney functions through increasing levels of urea and creatinin and decreasing levels of T.P and albumin significantly (P≤0.05). Moreover, all *C. tiglium* nano-extract treated groups showed significant (P≤0.05) decrease in levels of urea and creatinin significantly (P≤0.05) with restoring T.P and albumin levels to normal values.

*Tumor Markers*

**Figure 1:**
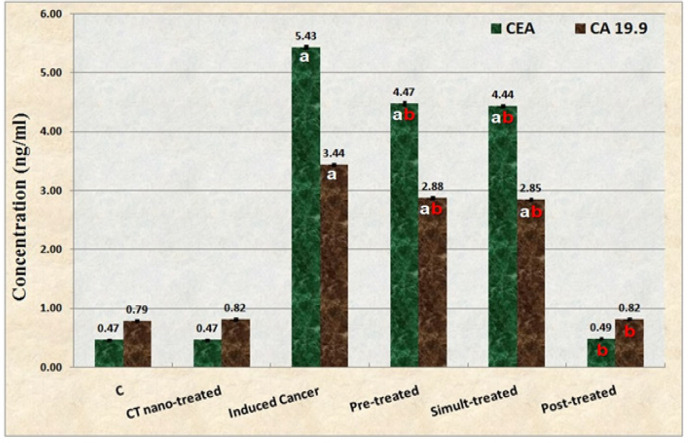
Data Showing the Ameliorative Effect of *C. tiglium* Seeds Extract after Incorporating Silver Nanoparticles (Ag-NPs) against Azoxymethane (AOM) Induced Colon Cancer on Tumor Markers in Sera of Rats

Data illustrated in Figure 1 showed that AOM caused significant (P≤0.05) elevation in levels of tumor markers (CEA and CA 19.9) as compared to control group. As compared to AOM induced colon cancer group, the treatment with *C. tiglium* nano-extract declined levels of those markers significantly (P≤0.05) in all nano-extract treated groups and restored their levels to normalcy in post-treated group.

*Inflammation Markers*

**Figure 2:**
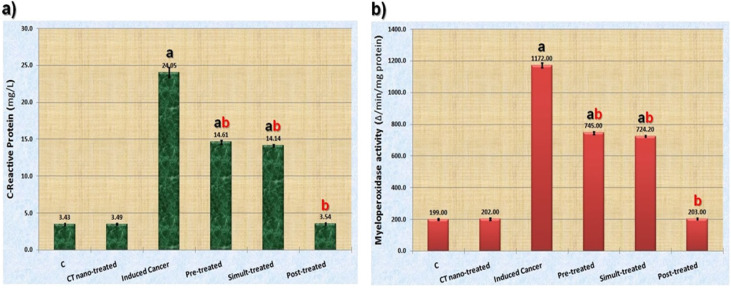
Data Showing the Ameliorative Effect of *C. tiglium* Seeds Extract after Incorporating Silver Nanoparticles (Ag-NPs) against Azoxymethane (AOM) Induced Colon Cancer on a) C-Reactive Protein (C-RP) in serum samples and b) Activity of Myeloperoxidase (MPO) in colon tissue homogenates of rats. Values expressed as mean ± SE; a, significant difference (P≤0.05) from control group; b, significant difference (P≤0.05) from toxic group

As regard to the inflammatory response, data illustrated graphically in Figure 2 showed that AOM caused significant (P≤0.05) elevation in levels of inflammatory markers (C-RP level and MPO activity) as compared to control group. 

Although the treatment with *C. tiglium* nano-extract caused significant (P≤0.05) decrease in levels of inflammatory markers in all nano-extract treated groups as compared to AOM induced colon cancer group, it restored levels of these measurements to normal values in post-treated group.

Major Colonic Contents and Oxidative Stress Markers

As regard to the assays that were carried out in the colon tissues, glucose and calcium in addition to LDH enzyme are considered as the major cellular contents in colon tissues. AOM caused significant (P≤0.05) decline in these measurements as compared to control group (Table 3).

Although the treatment with *C. tiglium* nano-extract caused significant (P≤0.05) increase in glucose level in all nano-extract treated groups as compared to AOM induced colon cancer group and restored its level to normalcy in post-treated group. On the other hand, it decreased activity of LDH as compared to AOM induced colon cancer group and could not restore its activity to normal value in all nano-extract treated groups. As regard to calcium level, *C. tiglium* nano-extract caused significant (P≤0.05) increase in its level with respect to AOM induced colon cancer group and restored its level to normalcy in all nano-extract treated groups.

Moreover, AOM caused significant (P≤0.05) decrease in level of the total antioxidant capacity (TAC) and activities of the antioxidant enzymes (CAT and GPx) associated with significant (P≤0.05) elevation in concentrations of the peroxidation products (LPO and TPC) as compared to control group as shown in Table 3. 

Although the treatment with *C. tiglium* nano-extract caused significant (P≤0.05) increase in TAC and activities of CAT and GPx associated with lowering concentrations of LPO and TPC in all nano-extract treated groups as compared to AOM induced colon cancer group, it restored these measurements to normal levels in post-treated group.

*Histopathological Examination and Immunohistochemistry*

**Figure 3:**
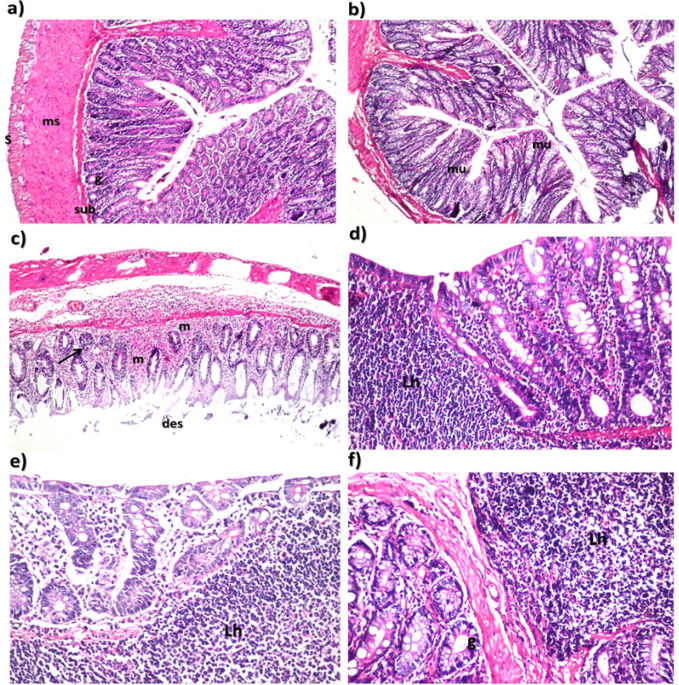
Histopathological Examination Showing the Ameliorative Effect of *C. tiglium* Seeds Extract after Incorporating Silver Nanoparticles (Ag-NPs) against Azoxymethane (AOM) Induced Colon Cancer on Colon Tissue of Rats. It revealed that a) control group appeared with normal histological structure of the mucosa with glandular structure (g), submucosa (sub), muscularis (ms) and serosa (S) (H and E, X40), b) *C. tiglium* nano-extract treated group appeared with normal histological structure (H and E, X16), c) AOM induced colon cancer group appeared with desquamation (des) of the tips in the mucosal lining epithelium with massive inflammatory cells infiltration in lamina propria of the mucosa and submucosa (m) as well as anaplasia of the glandular epithelium (red arrow) with criteria of malignancy of disorganization, hyperchromachia, polarity and pleomorphism with inflammatory cells infiltration in surrounding lamina propria (H and E, X16), d) *C. tiglium* nano-extract pre-treated group appeared with sever lymphoid hyperplasia (Lh) in the follicle submucosa with extension of mucosa (H and E, X40), e) *C. tiglium* nano-extract simult-treated group appeared with sever lymphoid follicles hyperplasia (Lh) in the submucosa and extended to the mucosa associated with inflammatory cells infiltration (m) (H and E, X40) and f) *C. tiglium* nano-extract post-treated group noticed with lymphoid follicles hyperplasia (Lh) in the submucosa with intact glandular structure (g) (H and E, X40).

As revealed in colon of control rats (Figure 3a), it was found that there was no histopathological alteration and the normal histological structure of the mucosa layer with lining epithelium and lamina propria with the glands as well as the underlying submucosa, muscularis and serosa were noticed. In *C. tiglium* nano-extract treated group (Figure 3b), there was no histopathological alteration and no deviation recorded from control group.

While, AOM induced colon cancer group, desquamation was detected in the tips of the lining mucosal epithelium while the underlying lamina propria of the mucosa and submucosa showed massive inflammatory cells infiltration (Moderate degree: ++ ; 50-75%) as well as anaplasia and dysplasia (Severe degree: +++ ; 75-100%) in the glandular epithelial structure as hyperchromachia, polarity and pleomorphism (Figure 3c).

In nano-extract pre-treated group, sever lymphoid follicles hyperplasia (Severe degree: +++ ; 75-100%) was detected in the submucosa and extended to the lamina propria of the mucosa (Figure 3d). In nano-extract simult-treated group, the submucosa showed severe lymphoid hyperplasia (Moderate degree: ++ ; 50-75%) in the follicles which was extended to the mucosa in association with inflammatory cells infiltration (Mild degree: + : 25-50%) in the later (Figure 3e). While in nano-extract post-treated group, the glandular structure was intact while the submucosa showed mild lymphoid hyperplasia (Mild degree: + : 25-50%) in the follicles (Figure 3f). The overall histological findings revealed that the *C. tiglium* nano-extract minimized severity of the inflammatory responses that is considered as criteria of malignancy and stage of colon cancer especially in the nano-extract post-treated group.

**Figure 4:**
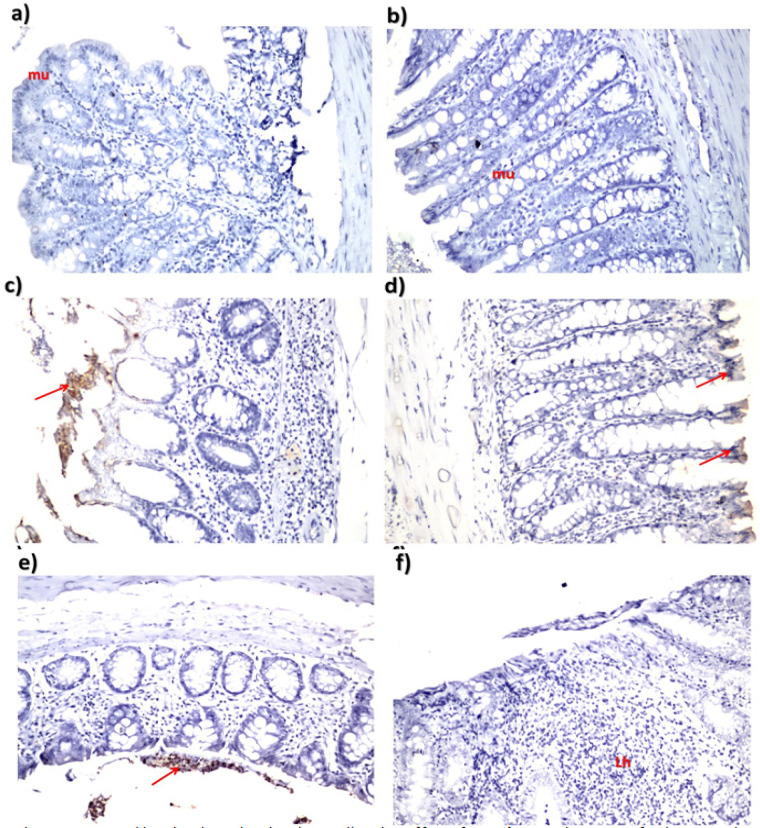
Immunohistochemistry Showing the Ameliorative Effect of *C. tiglium* Seeds Extract after Incorporating Silver Nanoparticles (Ag-NPs) against Azoxymethane (AOM) Induced Colon Cancer on Severity of the Immunohistochemical Reaction in Colon Tissue of Rats Using Anti-Keratin 20 in a) control group (H and E, X40), b) *C. tiglium* nano-extract treated group (H and E, X40), c) AOM induced colon cancer group (H and E, X40), d) *C. tiglium* nano-extract pre-treated group (H and E, X40), e) *C. tiglium* nano-extract simult-treated group (H and E, X40) and f) *C. tiglium* nano-extract post-treated group (H and E, X40).

On the other hand, the IHC was carried out for detection of the anti-Keratin 20 antibody through localization of the reaction on the tips of the mucosal epithelium. Severity of the immunoreactivity is depending on the density and distribution of the brown to black color. As presented in Figure 4, the anti-Keratin 20 antibody was not expressed in the control and *C. tiglium* nano-extract treated groups. While in the AOM induced colon cancer group, anti-Keratin 20 antibody was expressed severely (moderate degree: ++ ; 50-75%). Although the treatment with *C. tiglium* nano-extract could not prevent the presence of anti-Keratin 20 antibody completely in the pre-treated and simult-treated groups, it reduced its expression obviously (midl degree: + ; 25-50%). While in nano-extract post-treated group, it prevented expression of anti-Keratin 20 antibody completely.

*Electrophoretic Assays*

*Electrophoretic Protein Pattern*

**Figure 5:**
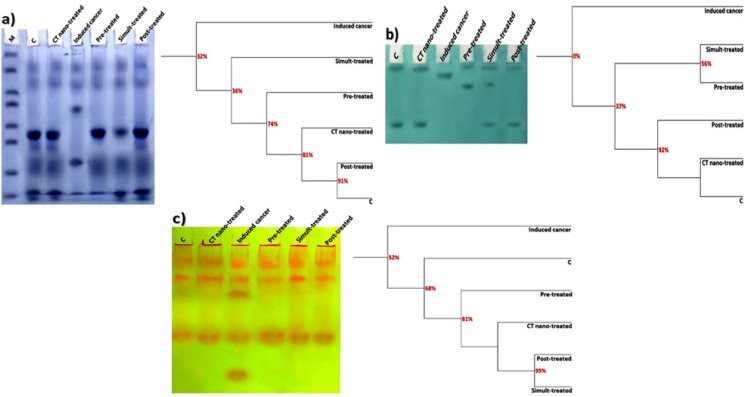
Native Electrophoretic Patterns Showing Physiological Effect of *C. tiglium* Seeds Extract after Incorporating Silver Nanoparticles (Ag-NPs) against Azoxymethane (AOM) Induced Colon Cancer on Bands Number, Arrangement and Similarity Percents on a) protein, b) lipid moiety of native protein and c) calcium moiety of native protein in colonic tissues of rats

As presented in Figure 5a, native protein pattern was represented electrophoretically in colon of control rats by 8 bands identified at Rfs 0.14, 0.24, 0.56, 0.64, 0.75, 0.78, 0.92 and 0.95 (Mwts 124.50, 74.26, 23.06, 19.37, 16.29, 15.39, 7.31 and 6.03 KDa; B% 8.88, 10.24, 15.38, 12.48, 13.37, 12.09, 14.50 and 13.05; Qty% 6.69, 7.17, 15.18, 4.70, 11.27, 7.59, 10.15 and 7.49, respectively). It was noticed that there were 5 common bands identified at Rfs 0.14, 0.24, 0.75, 0.92 and 0.95. *C. tiglium* nano-extract solely caused no alterations in the native protein pattern and no deviation detected from the control group. While AOM caused physiological alterations in the native protein pattern represented by hiding 3 normal protein bands with existence of the characteristic one that was identified in AOM induced colon cancer group at Rf 0.40 (Mwt 37.18 KDa; B% 18.77 and Qty% 11.76). Therefore, the lowest SI and highest GD values were recorded in AOM induced colon cancer group (SI=71.43% ; GD=28.57%).

Treatment with *C. tiglium* nano-extract exhibited improvement in the protein pattern by hiding the characteristic bands with restoring 2 normal bands identified at Rfs 0.55 and 0.78 (Mwts 23.88 and 15.44 KDa, B% 18.50 and 16.21 and Qty% 19.31 and 9.46, respectively) in nano-extract pre-treated group, one normal band identified at Rf 0.54 (Mwt 24.45 KDa, B% 17.95 and Qty% 15.23) in nano-extract simult-treated group and 3 normal bands identified at Rfs 0.55, 0.63 and 0.78 (Mwts 23.88, 19.80 and 15.53 KDa, B% 15.66, 12.51 and 13.92 and Qty% 16.45, 6.79 and 3.28, respectively) in nano-extract post-treated group. Therefore, SI values increased with decreasing GD values in the nano-extract pre-treated, simult-treated and post-treated groups (SI=93.33, 85.71 and 100.00% ; GD=6.67, 14.29 and 0.00%, respectively). Moreover, the relative quantities of the total protein bands declined significantly (P≤0.05) in AOM induced colon cancer group as compared to the control group (Table 4). Treatment with *C. tiglium* nano-extract increased the relative quantities significantly (P≤0.05) in all nano-extract treated groups with respect to AOM induced colon cancer group and restored quantities of the total bands to normal values. 

*Electrophoretic Lipid Moiety of Native Protein Pattern*

As revealed in Figure 5b, the native lipoprotein pattern was represented electrophoretically in colon of control rats by 2 bands identified at Rfs 0.03 and 0.32 (B% 47.68 and 52.32 and Qty% 6.94 and 7.13, respectively). There were no common bands. The *C. tiglium* nano-extract alone caused no alterations in the native protein pattern and no deviation detected from the control group. While it was noticed that AOM caused physiological alterations in the lipid moieties of native protein bands represented by hiding the normal bands with existence of characteristic (unique) one identified at Rf 0.07 (B% 100.00 and Qty% 6.06) in AOM induced colon cancer group. Therefore, the lowest SI and highest GD values were recorded in AOM induced colon cancer group (SI=0.00% ; GD=100.00%).

Treatment with *C. tiglium* nano-extract exhibited improvement in the lipoprotein pattern by restoring one normal band identified at Rf 0.02 (B% 49.02 and Qty% 2.69) in the nano-extract pre-treated group with existence of one abnormal band at Rf 0.12 (B% 50.98 and Qty% 6.49). It restored 2 normal bands identified at Rfs 0.03 and 0.32 (B% 32.22 and 34.48 and Qty% 4.23 and 5.11, respectively) in the nano-extract simult-treated group with existence of one abnormal band at Rf 0.12 (B% 33.30 and Qty% 10.01). While in nano-extract post-treated group, nano-extract restored all the normal bands at Rfs 0.03 and 0.32 (B% 49.47 and 50.53 and Qty% 3.61 and 5.53, respectively) without existence of characteristic ones. Therefore, the SI values increased with decreasing the GD values in the nano-extract pre-treated, simult-treated and post-treated groups (SI=66.67, 80.00 and 100.00%; GD=33.33, 20.00 and 0.00%, respectively).

As regard to the quantitative level, it was found that the relative quantities of the total lipoprotein bands declined significantly (P≤0.05) in AOM induced colon cancer group as compared to control group (Table 4). Although *C. tiglium* nano-extract increased the relative quantities significantly (P≤0.05) in all nano-extract treated groups with respect to AOM induced colon cancer group, relative quantities of the total bands restored to normal values in nano-extract post-treated group.

*Electrophoretic Calcium Moiety of Native Protein Pattern*

Data illustrated in Figure 5c showed that calcium moiety of native protein pattern was represented electrophoretically in colon of control rats by 3 common bands identified at Rfs 0.12, 0.23 and 0.64 (B% 35.00, 32.70 and 32.29 and Qty% 2.56, 9.33 and 1.93, respectively). *C. tiglium* nano-extract alone caused no alterations in the native protein pattern and no deviation detected from the control group. AOM caused physiological alterations in the calcium moieties of native protein bands represented by appearance of 2 characteristic (unique) bands identified at Rf 0.34 and 0.91 (B% 20.50 and 18.62 and Qty% 10.29 and 7.68, respectively) in AOM induced colon cancer group without hiding the normal ones. Therefore, the lowest SI and highest GD values were recorded in AOM induced colon cancer group (SI=75.00% ; GD=25.00%).

Treatment with *C. tiglium* nano-extract exhibited improvement in the calcium moiety of native protein pattern by hiding the abnormal characteristic bands in all nano-extract treated groups. Therefore, the SI values increased with decreasing the GD values in all the nano-extract treated groups (SI=100.00; GD=0.00%).

At the quantitative level, it was noticed that the relative quantities of the total bands increased significantly (P≤0.05) in AOM induced colon cancer group with respect to control group (Table 4). Treatment with *C. tiglium* nano-extract decreased the relative quantities significantly (P≤0.05) in all nano-extract treated groups with respect to AOM induced colon cancer group and restored quantities of the total bands to normal values. 

*Electrophoretic isoenzymes*

*Electrophoretic Catalase Pattern*

**Figure 6:**
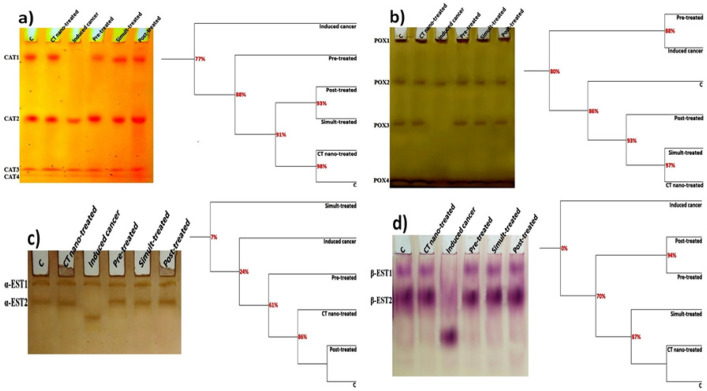
Native Electrophoretic Isoenzymes Patterns Showing Physiological Effect of *C. tiglium* Seeds Extract after Incorporating Silver Nanoparticles (Ag-NPs) against Azoxymethane (AOM) Induced Colon Cancer on Bands Number, Arrangement and Similarity Percents on a) Catalase (CAT), b) Peroxidase (POX), c) α-esterase (α-EST) and d) β-esterase (β-EST) in colonic tissues of rats

As illustrated in Figure 6a, it was noticed that CAT isoenzym pattern was represented electrophoretically in colon of control rats by 4 types identified at Rfs 0.11, 0.55, 0.91 and 0.95 (B% 26.55, 28.73, 24.72 and 19.99 and Qty% 12.35, 12.23, 4.53 and 2.88, respectively). Three CAT types (CAT2, CAT3 and CAT4) are considered as common bands. *C. tiglium *nano-extract alone caused no abnormalities or deviation from control group. AOM caused physiological changes in CAT pattern represented by hiding the CAT1 type without appearance of characteristic bands. Therefore, the lowest SI and highest GD values were recorded in AOM induced colon cancer group (SI=85.71% ; GD=14.29%). Treatment with *C. tiglium* nano-extract restored the physiological state of the electrophoretic CAT pattern through restoring the absent type (CAT1 type) in all nano-extract treated groups. Therefore, the SI values increased with decreasing the GD values in all the nano-extract treated groups (SI=100.00; GD=0.00%).

At the quantitative level, it was found that the relative quantities of the total CAT types decreased significantly (P≤0.05) in AOM induced colon cancer group as compared to control group (Table 4). Although treatment with *C. tiglium* nano-extract increased the relative quantities significantly (P≤0.05) in all nano-extract treated groups with respect to AOM induced colon cancer group, quantities of the total CAT types restored to normal values in the nano-extract post-treated group. 

*Electrophoretic Peroxidase Pattern*

As presented in Figure 6b, it was noticed that POX isoenzym pattern was represented electrophoretically in colon of control rats by 4 types identified at Rfs 0.02, 0.29, 0.56 and 0.94 (B% 23.73, 23.46, 23.59 and 29.22 and Qty% 4.73, 2.81, 3.29 and 4.47, respectively). Three POX types (POX1, POX2 and POX4) are considered as common bands. The *C. tiglium* nano-extract alone caused no abnormalities or deviation from control group. 

AOM caused physiological changes in the POX pattern represented by hiding POX3 type without appearance of characteristic ones. Therefore, the lowest SI and highest GD values were recorded in AOM induced colon cancer group (SI=85.71%; GD=14.29%). Treatment with *C. tiglium* nano-extract restored the physiological state of the electrophoretic POX pattern through restoring the absent type (POX3 type) in all nano-extract treated groups. Therefore, the SI values increased with decreasing the GD values in all the nano-extract treated groups (SI=100.00%; GD=0.00%).

As regard to the quantitative level, the relative quantities of the total POX types decreased significantly (P≤0.05) in AOM induced colon cancer group as compared to control group (Table 4). *C. tiglium* nano-extract decreased the relative quantities significantly (P≤0.05) in all nano-extract treated groups with respect to AOM induced colon cancer group and restored quantities of the total POX types to normal values.

*Electrophoretic Esterase Pattern*

As shown in Figure 6c, it was noticed that electrophoretic α-EST isoenzyme was represented in colon of control rats by 2 types identified at Rfs 0.04 and 0.15 (B% 50.58 and 49.42 and Qty 4.75 and 7.43, respectively). The first α-EST type (α-EST1) is considered as common band. *C. tiglium* nano-extract alone caused no deviation from control group. While AOM caused physiological changes in the α-EST pattern represented by hiding α-EST2 type without appearance of characteristic ones. Therefore, the SI value (SI=50.00%) decreased with increasing GD value (GD=50.00%) in AOM induced colon cancer group. 

Treatment with *C. tiglium* nano-extract restored the physiological state of the α-EST isoenzymes pattern through hiding the characteristic type with restoring the absent type (α-EST2 type) in all nano-extract treated groups. Therefore, the SI values increased with decreasing the GD values and became equal in all the nano-extract treated groups (SI=100.00% ; GD=0.00%).

As regard to the quantitative level, it was noticed that AOM caused no significant changes in the relative quantities of the total α-EST types and values of the relative quantities of total α-EST types were approximately equal in all treated groups as compared to control group and AOM induced colon cancer group (Table 4).

As presented in Figure 6d, it was noticed that electrophoretic β-EST isoenzyme was represented in colon of control rats by 2 types identified at Rfs 0.15 and 0.35 (B% 43.65 and 56.35 and Qty% 17.26 and 36.82, respectively). The second β-EST type (β-EST2) is considered as common band. *C. tiglium* nano-extract alone caused no deviation from control group. 

AOM caused abnormalities noticed physiologically in the β-EST pattern represented by hiding β-EST1 type with appearance of one characteristic (unique and abnormal) band identified at Rf 0.66 (B% 51.40 and Qty% 33.04). Therefore, AOM decreased the SI value with increasing the GD value (SI=50.00%; GD=50.00%) in AOM induced colon cancer group. The treatment with *C. tiglium* nano-extract restored the physiological state of β-EST isoenzymes pattern through hiding the characteristic type with restoring the absent type (β-EST1 type) in all nano-extract treated groups. Therefore, the SI values increased with decreasing the GD values and became equal in all the nano-extract treated groups (SI=100.00%; GD=0.00%).

At quantitative level, it was found that the relative quantities of the total β-EST types decreased significantly (P≤0.05) in AOM induced colon cancer group as compared to control group (Table 4). Treatment with *C. tiglium *nano-extract increased the relative quantities significantly (P≤0.05) in all nano-extract treated groups with respect to AOM induced colon cancer group and restored quantities of the total β-EST types to normal values. 

*Molecular assay*

**Figure 7:**
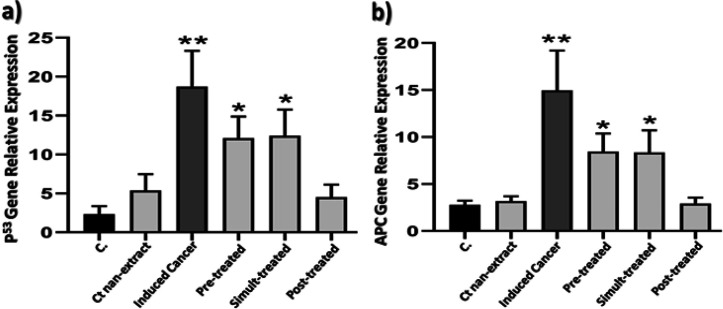
The Ameliorative Effect of *C. tiglium* Seeds Extract after Incorporating Silver Nanoparticles (Ag-NPs) Against the Deleterious Effect of azoxymethane (AOM) Induced Colon Cancer on a) level of Tumor Protein *P*^53^ (*TP*^53^) gene expression and b) level of *Adenomatous Polyposis Coli (APC)* gene expression in rats

As shown in Figure 7a, AOM induced colon cancer group showed highly significant increase (P=0.004) in level of *TP*^53^ gene expression as compared to control group. In addition, levels of *TP*^53^ gene expression are significantly higher in *C. tiglium* nano-extract pre-treated and simult-treated groups when compared to that of control group (P=0.026 and P=0.019, respectively). While the nano-extract post-treated group didn’t show any significant (P=0.417) change in level of *TP*^53^ gene expression than control group.

As illustrated in Figure 7b, the toxic group showed highly significant (P=0.006) increase in level of *APC* gene expression as compared to control group. In addition, levels of *APC* gene expression are slightly significantly (P=0.27 and P=0.020, respectively) higher in C. tiglium nano-extract pre-treated and simult-treated groups in comparison to control group. While the nano-extract post-treated groups didn’t show any significant (P=0.796) change in level of *APC* gene expression than control group.

**Table 1:** Effect of *C. tiglium *Seeds Extract after Incorporating Silver Nanoparticles (Ag-NPs) against Azoxymethane (AOM) Induced Colon Cancer on Different Hematological Measurements in Rats

		C	*C. tiglium *nano-extract	Induced cancer	*C. tiglium *nano-extract		
					Pre-treated	Simult-treated	Post-treated
Formed Elements	RBCs (10^6^/ul)	5.86 ± 0.18	5.94 ± 0.25	5.78 ± 0.19	5.92 ± 0.15	5.74 ± 0.16	5.82 ± 0.18
	HB (g/ dl)	12.13 ± 0.36	12.81 ± 0.29	12.34 ± 0.24	12.81 ± 0.22	12.77 ± 0.19	12.49 ± 0.22
	HCT (%)	33.32 ± 1.08	34.40 ± 1.94	32.29 ± 1.16	32.13 ± 0.58	32.25 ± 0.48	32.50 ± 0.48
	MCV (um^3^)	56.90 ± 1.02	57.00 ± 1.44	54.62 ± 1.02	55.73 ± 1.67	55.35 ± 1.15	54.94 ± 1.22
	MCH (pg)	20.97 ± 0.38	20.82 ± 0.29	21.29 ± 0.55	20.63 ± 0.43	20.79 ± 0.28	21.30 ± 0.47
	MCHC (g/ dl)	36.46 ± 0.41	35.78 ± 0.56	34.04 ± 0.98	34.26 ± 0.72	34.63 ± 0.85	34.31 ± 0.47
	RDW (%)	15.62 ± 0.39	15.64 ± 0.25	20.24 ± 0.18^a^	19.68 ± 0.27^a^	17.22 ± 0.17^ab^	15.54 ± 0.24^b^
	MPV (um^3^)	6.16 ± 0.15	6.21 ± 0.15	9.18 ± 0.10^a^	7.26 ± 0.11^ab^	7.65 ± 0.12^ab^	6.23 ± 0.14^b^
	PLT (10^3^/ul)	446.30 ± 5.64	450.00 ± 6.30	849.10 ± 11.48^a^	612.20 ± 8.46^ab^	512.00 ± 4.89^ab^	456.20 ± 7.02^b^
	WBCs (10^3^/ul)	8.71 ± 0.14	8.78 ± 0.15	15.13 ± 0.22^a^	12.81 ± 0.12^ab^	12.64 ± 0.11^ab^	8.99 ± 0.12b
Differential Count	Lymp. (10^3^/ul)	6.79 ± 0.13	6.75 ± 0.13	11.71 ± 0.16^a^	9.85 ± 0.13^ab^	9.82 ± 0.13^ab^	6.99 ± 0.11^b^
	Mono. (10^3^/ul)	1.00 ± 0.06	0.98 ± 0.06	1.72 ± 0.07^a^	1.63 ± 0.09^a^	1.21 ± 0.04^b^	1.03 ± 0.02^b^
	Gran. (10^3^/ul)	0.94 ± 0.03	0.91 ± 0.01	1.91 ± 0.02^a^	1.57 ± 0.02^ab^	1.24 ± 0.02^ab^	0.89 ± 0.02^b^

Values were expressed as mean ± standard error, ^a^, significant difference from control group; ^b^, significant difference from toxic group at P≤0.05.

**Table 2:** Effect of *C. tiglium* Seeds Extract after Incorporating Silver Nanoparticles (Ag-NPs) against Azoxymethane (AOM) Induced Colon Cancer on Different Biochemical Measurements in Rats

		C	*C. tiglium* nano-extract	Induced cancer	*C. tiglium *nano-extract		
					Pre-treated	Simult-treated	Post-treated
Liver Enzymes	ALT (U/L)	44.70 ± 0.83	45.60 ± 1.10	84.70 ± 0.83^a^	44.10 ± 0.82^b^	45.49 ± 0.64^b^	44.50 ± 1.01^b^
	AST (U/L)	173.57 ± 1.47	170.60 ± 2.46	268.00 ± 3.12^a^	169.90 ± 1.31^b^	169.20 ± 1.88^b^	167.90 ± 1.73^b^
	ALP (U/L)	84.00 ± 0.72	83.30 ± 0.73	131.30 ± 1.26^a^	84.10 ± 0.61^b^	83.30 ± 0.68^b^	84.00 ± 0.78^b^
Lipid Profile	TC (mg/dl)	75.70 ± 0.68	75.20 ± 0.89	96.10 ± 0.64^a^	74.20 ± 0.61^b^	75.30 ± 0.67^b^	74.70 ± 0.78^b^
	T.Gs (mg/dl)	64.61 ± 0.67	65.39 ± 0.83	93.52 ± 0.57^a^	64.55 ± 0.75^b^	64.65 ± 0.64^b^	64.44 ± 1.12^b^
kidney functions	Urea (mg/dl)	57.56 ± 1.19	58.11 ± 1.24	102.671 ± 1.75^a^	59.20 ± 1.15^b^	58.83 ± 1.02^b^	58.97 ± 1.12^b^
	Creatinin (mg/dl)	0.81 ± 0.01	0.81 ± 0.02	2.57 ± 0.04^a^	0.794 ± 0.01^b^	0.80 ± 0.01^b^	0.81 ± 0.01^b^
	Total Protein (g/dl)	8.48 ± 0.07	8.49 ± 0.09	4.91 ± 0.07^a^	8.43 ± 0.11^b^	8.38 ± 0.08^b^	8.36 ± 0.13^b^
	Albumin (g/dl)	3.43 ± 0.07	3.50 ± 0.08	1.27 ± 0.05^a^	3.43 ± 0.07^b^	3.44 ± 0.10^b^	3.46 ± 0.08^b^

Values were expressed as mean ± standard error, ^a^, significant difference from control group; ^b^, significant difference from toxic group at P≤0.05.

**Table 3:** Effect of *C. tiglium* Seeds Extract after Incorporating Silver Nanoparticles (Ag-NPs) against Azoxymethane (AOM) Induced Colon Cancer on the Major Cellular Contents and Oxidative Stress Markers in Colon Tissues of Rats

		C	*C. tiglium *nano-extract	Induced cancer	*C. tiglium* nano-extract		
					Pre-treated	Simult-treated	Post-treated
Major Cellular Contents	Glucose (nmol/ g wet tissue)	3.70 ± 0.04	3.70 ± 0.07	1.13 ± 0.02^a^	1.79 ± 0.02^ab^	2.58 ± 0.03^ab^	3.73 ± 0.02^b^
	LDH (U/ g wet tissue)	1742.41 ± 6.08	1737.00 ± 4.64	1047.80 ± 11.24^a^	1411.40 ± 20.51^ab^	1412.40 ± 14.08^ab^	1552.98 ± 26.67^ab^
	Ca (mmol/ g wet tissue)	0.17 ± 0.01	0.18 ± 0.00	0.96 ± 0.01^a^	0.15 ± 0.01^b^	0.17 ± 0.00^b^	0.15 ± 0.01^b^
Markers of Oxidative Stress	TAC (mM/L)	1.31 ± 0.04	1.27 ± 0.07	0.48 ± 0.01^a^	0.83 ± 0.01^ab^	0.85 ± 0.01^ab^	1.29 ± 0.05^b^
	CAT (U/gm)	45.25 ± 0.24	45.29 ± 0.81	10.37 ± 0.14^a^	31.74 ± 0.24^ab^	33.81 ± 0.32^ab^	44.47 ± 0.23^b^
	GPx (U/gm)	21.37 ± 0.14	21.74 ± 0.28	11.40 ± 0.17^a^	17.08 ± 0.27^ab^	16.41 ± 0.26^ab^	20.39 ± 0.37^b^
	LPO (nmol/ g wet tissue)	44.00 ± 0.89	43.80 ± 0.58	144.40 ± 1.40^a^	75.40 ± 0.51^ab^	75.00 ± 0.95^ab^	43.40 ± 1.12^b^
	TPC (nmol/ mg Protein)	10.43 ± 0.06	10.36 ± 0.06	24.14 ± 0.36^a^	18.08 ± 0.26^ab^	13.94 ± 0.12^ab^	10.74 ± 0.21^b^

Values were expressed as mean ± standard error; a, significant difference from control group; b, significant difference from toxic group at P≤0.05.

**Table 4:** Effect of *C. tiglium* Seeds Extract after Incorporating Silver Nanoparticles (Ag-NPs) against Azoxymethane (AOM) Induced Colon Cancer on Relative Quantities of the Total Bands of Electrophoretically Separated Proteins and Isoenzymes Patterns in Colonic Tissues of Rats

		C	*C. tiglium * nano-extract	Induced cancer	*C. tiglium* nano-extract		
					Pre-treated	Simult-treated	Post-treated
Proteins Patterns	Native protein	70.81 ± 0.48	72.26 ± 1.17	42.39 ± 0.72^a^	68.46 ± 0.43^b^	69.92 ± 0.28^b^	71.67 ± 0.72^b^
	Lipid moiety of native protein	13.96 ± 0.06	13.33 ± 0.40	5.95 ± 0.04^a^	9.81 ± 0.02^ab^	18.99 ± 0.18^ab^	12.93 ± 0.30^b^
	Calcium moiety of native protein	13.79 ± 0.19	13.85 ± 0.23	34.11 ± 0.11a	13.93 ± 0.11^b^	13.85 ± 0.13^b^	13.82 ± 0.29^b^
Isoenzymes Patterns	CAT	31.94 ± 0.12	31.94 ± 0.14	16.54 ± 0.28^a^	27.63 ± 0.25^ab^	27.64 ± 0.29^ab^	32.14 ± 0.30^b^
	POX	15.68 ± 0.24	15.61 ± 0.24	9.01 ± 0.10^a^	15.52 ± 0.34^b^	15.70 ± 0.27^b^	15.51 ± 0.26^b^
	α-EST	12.58 ± 0.18	12.45 ± 0.27	12.42 ± 0.29	12.43 ± 0.34	12.39 ± 0.14	12.37 ± 0.25
	β-EST	54.46 ± 0.31	53.37 ± 0.45	31.53 ± 0.52^a^	52.40 ± 0.37^b^	52.46 ± 0.37^b^	52.64 ± 0.40^b^

Values were expressed as mean ± standard error; a, significant difference from control group; b, significant difference from toxic group at P≤0.05

## Discussion

Although the present study was designed to reveal efficiency of *C. tiglium* extract after incorporating Ag-NPs against colon cancer induced by AOM, it was found that AOM caused different deleterious effects at different hematological and biochemical levels during the induction process. It is necessary to use animal models for studying pathogenesis of colorectal tumors, as well as for evaluating the possible alternative treatments (De-Souza and Costa-Casagrande, 2018). Assessment of the haematological parameters can be used to determine the adverse effect on blood constituents of an animal and also it can be used to explain blood relating functions of chemical compounds/plant extract (Ashafa et al., 2009). 

It is well known that AOM induced alterations in haematological status of rats and this attributed mainly to the oxidative stress that plays a role in haematological abnormalities and carcinogenesis progression (Childress, 2012). During the current study, it was found that level of RDW elevated significantly in AOM induced colon cancer. This was in agreement with Forhecz et al., (2009) who reported that the RDW level elevated in active malignancy that is accompanied by a prolonged inflammatory processes. In addition, the RDW value is closely related to colorectal cancer metastasis and it has been shown to predict advanced cancer stage (Lee et al., 2014 ; Yang et al., 2018). The MPV level is considered as an early marker of platelet activation. It elevated significantly in AOM-induced colon cancer. This was in agreement with Li et al., (2014) who reported that MPV was found to be independently associated with the presence of colon cancer and there was a positive correlation between MPV and stage of tumor-nodule-metastases. The PLT level elevated significantly in addition to WBCs and its differential count (Lymp., Mono. and Gran.) in AOM induced colon cancer group. This might be attributed to the inflammatory reactions which stimulate the bone marrow to produce PLT and leukocytes especially lymphocytes because the leukocytes migrate to inflammation site and their attraction are facilitated by PLT (Laoui et al., 2011; Vieira-de-Abreu et al., 2012). Moreover, neutrophils are considered as the first line of defense and rapidly recruited to inflamed loci in response to inflammatory mediators released at the injury site (Coussens and Werb, 2002). Recent studies revealed that AOM can induce hepatotoxicity and develop liver injury (Megaraj et al., 2014), similarly to results of the current study. It was found that AOM caused elevation in levels of ALP, AST and ALP that are considered as the most common biomarkers used to evaluate liver functions. This was in accordance with Moharib et al., (2014) who reported that AOM caused liver damage occurred as a result of highly reactive electrophiles (carbonium ions and alkyl free radicals) that severely damage liver tissue causing necrosis and consequently leads to loss of membrane integrity. Therefore, cytoplasmic enzymes might leak from hepatocytes into blood at early stages of liver damage due to increasing membrane permeability.

Silver *C. tiglium* nano-extract restored these measurements to normalcy at all therapeutic modes (Protection and Treatment). This might be attributed to the presence of wide range of the polyphenolic compounds that increased after incorporating Ag-NPs as suggested by Abdelhady and Badr (2016) and supported by Aboulthana et al., (2019) who revealed that *C. tiglium* nano-extract contains higher concentrations of the active ingredients that exhibit antioxidant activity when compared to the crude one. Consequently, these compounds exhibited high ability to maintain integrity of cell membranes against reactive oxygen species (ROS) generated as a result of AOM injection.

It was found that AOM caused alterations in kidney functions. This was in agreement with Garba et al., (2007) who postulated that serum urea and creatinin were measured as indicators of kidney function and raising their concentrations are considered as an index of kidney dysfunction. Furthermore, it is well known that AOM is an active metabolite of the colon specific carcinogen 1,2-dimethylhydrazine (DMH) and it belongs to the hydrazine compounds which stimulate urea cycle and up-regulate biosynthesis of amino acids (Liao et al., 2012). Therefore, urea cycle might be expected to be up-regulated to remove excess ammonia derived from elevated amino acids. Also, amino groups (N-terminus) of amino acids that have been used as metabolic fuel are converted into urea through urea cycle resulting in increase of urea level (Bando et al., 2011). As regard to decline in the TP, significant increase in urea and creatinin levels act as an inhibitor of the synthesis of proteins (Yazar and Baydan, 2008). Moreover, levels of TP and albumin might be decreased significantly as a result of the liver injury that consequently leads to alterations in protein biosynthesis (Gürocak et al., 2013). In addition, urea and creatinin levels might be increased with lowering TP and albumin levels due to the severe inflammation occurred in kidney tissues as a result of AOM injection and this consequently leads to tubules degeneration and protein cast deposition within tubular lumina and tissue necrosis (Tan et al., 2015).

Treatment with *C. tiglium* nano-extract restored levels of the kidney functions to normal levels at all therapeutic modes. This might be attributed to presence of high concentrations of flavonoids that improve the renal physiology and possess diuretic and natriuretic properties, as well as exerting renoprotective effects that attributed to antiapoptotic and antifibrotic properties of these active constituents. In addition, levels of the TP and albumin restored to normal values due to the beneficial effect of nano-extract on liver tissues that became able to biosynthesize protein normally (Vargas et al., 2018). 

Lipids play a crucial role in maintaining the membrane integrity (Chung and Jung, 2003). In the present study, it was found that AOM caused significant elevation in levels of TC and T.Gs. This was in accordance with Yuvaraj et al., (2007) who emphasized that chemical agents that induce cancer development affect cellular lipids and cause abnormal lipid synthesis or defective degradation of lipids. Also, this might be attributed to deficiency of the lipoprotein lipase that helps in uptake of the T.Gs (Lanza-Jacoby, 1984). In addition, Nalini et al., (2006) postulated that hypertriglyceridemia occurred as a result of tumors growth and it showed a stronger association with metastatic conditions. *C. tiglium* nano-extract decreased TC and T.Gs levels and this might be attributed to efficiency of the active constituents in the extract to develop lipolysis-stimulating agent in adipocytes (Kim et al., 2014). The lipolytic action of the extract was mainly mediated by phosphorylation of hormone-sensitive lipase (the key enzyme in the mobilization of fatty acids in adipocytes) and its activity is regulated post-transcriptionally by reversible phosphorylation by protein kinase A and this might aid in development of therapeutic strategy in preventing obesity (Holm, 2003).

It is well known that CEA and CA19-9 are the most common and specific markers of colon cancer. Levels of these markers are used not only for preoperative assessment of extent and outcome of cancer, but also postoperative monitoring of recurrence (Nakatani et al., 2012). Their levels are considered as the best serum marker for CRC and most thoroughly characterized tumor-associated antigens, in both biochemical and clinical aspects (Ogata et al., 2009). In the present study, it was found AOM caused significant elevation in levels of these tumor markers. This was in accordance with many previous studies carried out by Mare et al., (2013) who postulated that AOM was categorized as a potent carcinogen and active intermediate of DMH and it leads to generation of ROS in the colon and hence, induce tissue lesions. Consequently, these ROS cause instability of colon cell metabolism leading to different changes in these tumor markers that are categorized as representatives of colon function. Moreover, elevation of serum CEA and CA 19-9 levels was significantly correlated with larger lesion size and multiplicity of adenomas (Kim et al., 2017). Treatment with *C. tiglium* nano-extract decreased levels of the tumor markers and this might refer to the anti-tumor efficiency of the active constituents that increased after incorporating Ag-NPs in *C. tiglium* extract (Aboulthana et al., 2019). These active constituents exhibited higher free radicals scavenging activity, reducing power and total antioxidant capacity that can inhibit the process of carcinogenesis effectively and prevent the development of invasive cancer. Therefore, they exhibit anti-tumor activity through inhibiting growth of cancer cells (Mohd Ali et al., 2012 ; Abdelhady and Badr, 2016).

The C-RP is called positive protein of the acute phase. Its level increases dramatically as a marker of tissue damage, inflammation and associated with prognosis in a variety of solid tumors (Hall et al., 2013). In the present study, the C-RP level elevated significantly in AOM induced colon cancer group. This might reflect ongoing inflammation and tissue damage more accurately than other biochemical measurements (Masoodi et al., 2009). Furthermore, Rajendiran et al., (2018) emphasized that elevating C-RP level might be attributed to the inflammatory effect of DSS that ingested via drinking water in combination with AOM during the induction process. It is known that MPO is an enzyme found predominantly in neutrophils and it is used as a good index of inflammatory response and tissue injury. Its activity is linearly related to neutrophil infiltration in colonic tissue (DOdorico et al., 2001). During the present study, it was found that the MPO activity is significantly higher in AOM injected group. This might refer to neutrophil infiltration that increased as a result of ingestion of DSS in association with AOM (Pandurangan et al., 2015). Treatment with *C. tiglium* nano-extract decreased levels of the inflammatory markers. This is in accordance with Jangid and Jadhav (2018) who documented that the anti-inflammatory effect of this plant extract against intestinal inflammation might be attributed to presence of the secondary metabolites that are well known by their ability to inhibit inflammatory reactions.

LDH belongs to the most important enzymes that play an effective role in the glycolytic pathway. It catalyzes conversion of pyruvic acid anaerobically and reversibly into lactic acid. It is used as an indicator for cancer development and also for many abnormalities in different tissues (Erez et al., 2014). During the current study, it was found that glucose level decreased significantly as well as, decline in LDH activity in the AOM induced colon cancer group. This was in agreement with Zhao et al., (1997) who postulated that glycolytic pathway increases in cancer tissue. This might be attributed to increasing number of cells that consume great glucose amount to get energy through glycolysis during cancer development and hence LDH activity consumed under anaerobic conditions (Denko, 2008). Moreover, growing cancer cells are able to destroy other tissues causing release of intracellular enzyme like LDH into blood stream by injury or death of cells and hence LDH decreased in cancer tissue (Pujara and Chaudhary, 2017). Also, calcium plays a primary and effective role in development of cell injury in vital organs at different pathological states. During the current study, calcium level increased in colon tissues of AOM induced colon cancer group. This was in accordance with Wulf (2000) who reported that calcium level increased as a result of the oxidative stress and disturbances of calcium homeostasis that might be induced in three different subcellular compartments including cytoplasm, mitochondria and the endoplasmic reticulum. Furthermore, marked elevation in level of cytoplasmic calcium was related to injury of colon tissues during the induction process. Nano-extract restored the glucose level, LDH activity and calcium level to normal values. This was attributed to efficiency of the active constituents to inhibit several metabolic intermediates and ROS formed during microsomal enzyme activation process (Waly et al., 2014). 

The MDA is considered as one of the final polyunsaturated fatty acids peroxidation products that are commonly known as marker of oxidative stress (Al-Henhena et al., 2015). AOM exhibited significant increase in the MDA concentration in colon tissues. This was in accordance with Khan and Sultana (2011) who reported that elevation of the colonic MDA exhibited mutagenic and genotoxic effect and may contribute to cancer development. Also, it induced protein damage, DNA fragmentation, gene mutations and loss of membrane integrity (Anilakumar et al., 2010). Therefore, lipid and protein peroxidation products (LPO and TPC) increased significantly in AOM induced colon cancer group during the induction process of colon cancer. Antioxidant enzymes (CAT and GPx) provide the first line of cellular defense against the destructive effects of ROS. In the present study, it was noticed that AOM caused significant decline in total antioxidant capacity and activities of these antioxidant enzymes. This was in accordance with Waly et al., (2016) who emphasized that AOM has the ability to initiate oxidative stress through producing extremely ROS and by modifying activities of the antioxidant enzymes. Furthermore, AOM induced the oxidative stress through depletion of intracellular reduced glutathione (Lai et al., 2013). The plant extract contains wide range of the active compounds which exhibit antioxidant properties in a dose dependent manner (Popov et al., 2011). Moreover, due to the phenolic compounds available in this extract leading to antioxidant activity, it can scavenge of free radicals such as hydroxyl radical which is the major cause of peroxidation reactions (Ahmed, 2010). The colonic antioxidant profile was restored to normal levels by *C. tiglium* nano-extract. This might be attributed to nano-extract efficiency to prevent ROS generation in the colinic cells as presented by its ability to quench and scavenge DPPH and ABTs radicals’ formation (Waly et al., 2014). Dhanapal and Ping (2017) supported our findings and revealed that the antioxidant capacity of the plant extract increased after incorporating Ag-NPs. Due to its preference to adsorb antioxidant compounds onto surface of the Ag-NP, improving the scavenging ability.

Conventionally, carcinogenesis is well defined by three stages: initiation, promotion and progression (Martín et al., 2016). In the present study, the H and E staining of colonic tissues was carried out for histopathological examination and it was found that severe histological abnormalities were noticed in colonic mucosa of the AOM-treated rats. This was in accordance with Saleem et al., (2015) who emphasized that oxidative stress and depletion of the antioxidant enzymes were responsible for inflammatory cells infiltration and severe histopathological alterations noticed in AOM induced colon cancer group. Moreover, AOM is a metabolite of DMH, whose mechanism of preneoplastic lesions induction is caused by the increased expression of c-fos gene and reduced c-myc gene expression, as well as alterations in the mutated K-ras gene expression similar to those observed in spontaneous carcinogenesis in humans (Caderni et al., 2003). Also, alterations in architecture of colonic mucosa with typical inflammatory changes such as crypt and surface epithelial loss as well as infiltration of inflammatory cells and complete destruction of the epithelial architecture might refer to the effect of DSS that administrated via drinking water in association with AOM during the induction process (Huang et al., 2010). 

In addition, cytokeratin 20 was expressed severely in colonic tissues of AOM induced colon cancer. This was in accordance with Derry et al., (2014) who showed that cytokeratin 20 is considered as an additional colon biomarker and expressed virtually in epithelial cells in all cases of colorectal carcinomas. The present study revealed that *C. tiglium* nano-extract minimized severity of the inflammatory responses that is considered as criteria of malignancy and stage of colon cancer in nano-extract simult-treated group while these inflammatory responses were prevented completely in the post-treated group. Due to presence of phenolic compounds that are considered as part of a complex defense mechanism against wide range of stressors, they accumulate in response to these deleterious factors (Kumazawa et al., 2002). These phenolic compounds are well known as good antioxidant agents due to their capability of singlet oxygen-quenching and radical scavenging activity and incorporation of Ag-NPs into the extract increased the antioxidant efficiency (Mitiku and Yilma, 2017; Shousha et al., 2019). Herein, treatment with nano-extract protected the colonic cells from damage and dysfunction that induced by oxidative stress. This might refer to the beneficial efficiency of *C. tiglium* nano-extract to protect cells by maintaining membrane permeability through preventing glutathione depletion and hence inhibiting peroxidation reactions.

Electrophoresis is standard technique for separating, identifying and quantifying of different proteins and it is considered as the most commonly tool used to analyze stoichiometry of a specific subunit of a protein complex (Shah et al., 2010). Mutations may occur at qualitative level by disappearance of normal bands and appearance of one or more abnormal ones. Otherwise, the alterations may occur quantitatively by remaining normal bands but with altering their quantities. The SI is only correlated to the qualitative alterations (Aboulthana et al., 2016). The SI is inversely proportional to the genetic variation. The low SI values between control and all the treated groups indicate differences in bands number and arrangement (Abdalla et al., 2015 ; Sharada et al., 2015).

Proteins represent the key players inside the cells. They are susceptible to be oxidized and this depends on relative content of oxidation-sensitive amino acid residues (Östman et al., 2011). In the present study, AOM caused alterations in native protein pattern. This was in agreement with Yasui and Tanaka (2009) who suggested that the alterations in native protein might be attributed to interaction of AOM with proteins. Furthermore, Aguilar-Mahecha et al. (2002) reported that the electrophoretic alterations might refer to deleterious effect on RNA transcripts, altering their amounts, localization or translation. Also, the modifications in overall native protein pattern might be related to altering DNA organization and hence changing DNA function and hence protein expression (Harrouk et al., 2000). Lipids are the most sensitive part of the cellular macromolecules (Javed et al., 2014). They are the major components of chromosomes, chromatin and nuclear matrix (Struchkov et al., 2002). During the present study, AOM altered the native lipoprotein pattern severely. This might refer to depletion of the antioxidant defenses and accumulation products of the oxidative stress that attack the lipid portion through inducing formation of the ROS as suggested by El-Sayed et al., (2018). Consequently, this leads to oxidative modifications and denaturation of the lipid moieties of proteins (Blasiak et al., 2004). Calcium-binding proteins categorized as acidic proteins with low molecular weights. They exhibited inhibitory effect hydroxyapatite formation leading to alterations in the mineralization process (Hunter et al., 1996). Furthermore, they exhibit an effective role in resistance of the tissues against the toxic agents (Surowiak et al., 2007). The alterations in these proteins might occur as a result of abnormal tissue mineralization (Abulyazid et al., 2017). During the current study, it was noticed that AOM caused changes in calcium moieties of the native protein. This was in agreement with El-Sayed et al., (2018) who postulated that these qualitative and quantitative abnormalities in calcium moieties of the native protein might be attributed to the oxidative stress and generation of ROS that convert an active hydrogen atom from these biologically active macromolecules. Furthermore, these alterations might be related to the significant increase in calcium concentration in the colon tissue as described during the current experiment and this leads to stimulating the oxidative stress and hence leads to variations in this protein pattern. *C. tiglium* nano-extract showed ameliorative effect against the alterations induced by free radicals’ attack in different native electrophoretic protein patterns. This might refer to presence of the active polyphenolic constituents that increased as a result of incorporating Ag-NPs as reported by Aboulthana et al., (2019).

Antioxidant enzymes are considered as tissue dependent tools of the antioxidant system. Their activities vary from tissue to tissue. The changes in antioxidant system might refer to attack of the ROS targeting protein contents and altering metabolic pathways (Ramanathan et al., 1999). In the current study, AOM caused abnormalities in the electrophoretic antioxidant isoenzymes. This was in agreement with Aboulthana et al., (2016) who suggested that changing the fractional activity of different isoenzymes seemed to be correlated with the changes in rate of protein expression secondary to DNA damage induced by ROS. Consequently, this leads to structural changes in protein portion of native enzymes (Abulyazid et al., 2017). No changes occurred in the enzymatic activities, if there were no alterations in the protein expression (Djordjevic et al., 2010). Furthermore, these alterations might be related to the significant decrease in the glucose level and LDH activity in the colon tissue as described during the current experiment and this referred to altering the glycolytic pathway and hence changing the glycation process that consequently inhibits enzyme activity (Hook and Harding, 1998). Moreover, AOM altered the electrophoretic CAT pattern. This might be attributed to binding of this toxic substance to native macromolecules inducing secondary structural changes in the enzyme (Mansouri et al., 2013). Also, the alterations in the electrophoretic POX pattern might refer to the oxidative stress and the peroxidation reactions (Codrington et al., 2007). *C. tiglium* nano-extract showed antioxidant efficiency against the alterations in electrophoretic CAT and POX patterns. This might be due to enhancing the polyphenolic compounds that exhibit scavenging activity against ROS attack (Kumazawa et al., 2002).

Esterase-like albumin activity used as prognostic marker for various choronic diseases (Thangthaeng et al., 2011). It is expressed as several molecular forms that can be distinguished by their molecular weights and hydrodynamic properties (Massoulie et al., 1993). It can be visualized by staining the substrate using α- and β-naphthyl acetate in the presence of Fast Blue RR salt as a dye coupler (Ahmad et al., 2012). The alterations in electrophoretic EST pattern might be due to enhancement of oxidative stress markers which consequently results in altering albumin structure and function (Gangadharan et al., 2007). Also, the characteristic changes in EST pattern might refer to changing glycosylation of different ESTs in bile duct ligation liver. This consequently reflects changes in the glycosylated EST forms. The molecular EST pattern that remains unaffected, displays normal glycosylation pattern (Gravel et al., 1996). The glycosylation process that occurred abnormally increases probability of protein degradation and affected EST stability (Saxena et al., 1997). Due to increasing concentration of the total polyphenols after incorporating Ag-NPs, nano-extract exhibit antioxidative properties that could play an important role in maintaining integrity of macromolecules against oxidative reactions (Fraga et al., 2010). In addition, these polyphenols stimulate activity of antioxidant enzymes to overcome attack of free radicals targeting these biomacromolecules (Ashokkumar and Sudhandiran, 2008).

Cancer progression is usually associated with irregularities in cell cycle (Park and Lee, 2003). Recent study directed to search in nature to find evidence supported experimentally suppression of the concomitant involvement of cell cycle by mean of apoptosis to phytochemicals with cell-cycle modulatory effects (Moghadamtousi et al., 2014). The *TP*^53^ and *APC* are the most sensitive genes susceptible to harbor mutations induced colorectal cancers involving the distal colon (Lynch and de la Chapelle, 2003). In the current study, it was found that AOM induced colon cancer caused significant elevation in relative expression of *TP*^53^ gene and this was in agreement with Nambiar et al., (2002) who reported that level of *TP*^53^ gene expression might be attributed to increase accumulation of *TP*^53^ protein within the tumor cells. Moreover, it was suggested that elevation of the *TP*^53^ gene expression might refer to stability of *TP*^53^ protein that increased as a result of the aberrant alterations in *TP*^53^ acetylation and/or phosphorylation status and this suppresses its binding to DNA consensus sequences. Also, transactivation and repression function of *TP*^53^ protein might be inhibited by alternative mechanisms. Suppression of *TP*^53^ activity in AOM-induced colon cancer may refer to differences in its post-translational modification (Minamoto et al., 2001). This was supported by Nambiar et al., (2002) who illustrated that *TP*^53^ protein isolated from AOM-induced colon tumors was found to migrate more slowly on gel relative to the active form of *TP*^53^ isolated from normal cells. *APC* exhibits its normal functions by promoting cellular differentiation of intestinal lineages including mucus-producing goblet cells (Barker et al., 2009). It is considered as one of the tumor suppressor genes which are susceptible to various mutations (Femia et al., 2007). It is the most frequent mutated gene expressed as a result of precancerous lesions in colon carcinogenesis (Xu et al., 2017). 

During the current study, it was noticed that AOM caused significant elevation in relative expression of *APC* gene and this was supported by the results obtained by Grivennikov et al., (2012) who reported that level of APC gene expression was noticed significantly greater in AOM induced colon cancer group. This attributed to role of APC that acts as a central gatekeeper protein in colorectal tumorigenesis for regulating multiple cancer pathways. The alterations in expression of *APC* gene lead to changes in expression of APC protein and abnormalities in cell cycle and hence promoting colon cancer (Ghatak et al., 2017).

Treatment with *C. tiglium* nano-extract decreased level of the relative expression of *TP*^53^ and *APC* genes in the post-treated group to normal level. Although it could not decrease their levels in the pre-treated and simult-treated groups to normalcy, it decreased their levels significantly as compared to AOM-induced cancer. This was in accordance with the results obtained by Li et al., (2016) who verified that extract induced cellular apoptosis through regulating the apoptotic indexes, apoptotic rates and apoptosis-associated gene expressions. The extract is rich in the croton alkaloid and diterpenoids that have been found to inhibit cell proliferation or induce programmed cell death to control the growth of malignant cells (Thuong et al., 2014). Concentrations of these phyto-constituents increased after incorporating Ag-NPs (Aboulthana and Sayed, 2018; Aboulthana et al., 2019; Shousha et al., 2019). Consequently, the cytotoxic efficiency of nano-extract against growth of human colon cancer cells increased in comparison with the crude ones. Therefore, efficiency of *C. tiglium* extract was evaluated as a new plant-derived anticancer agent after incorporating silver nanoparticles during the current study.

In conclusion, the study concluded that *C. tiglium *nano-extract exhibited ameliorative effect against AOM induced colon cancer by decreasing levels of the hematological and biochemical measurements and restored their levels to normal values in all nano-extract treated groups. It restored levels of the tumor (CEA and CA 19-9) and inflammatory (C-RP and MPO) markers to normalcy in post-treated group. Moreover, it improved the antioxidant system by increasing activities of the antioxidant enzymes (CAT and GPx) with decreasing products of the peroxidation reactions (LPO and TPC) in all nano-extract treated groups and restored their levels to normal values in post-treated group. It minimized severity of the inflammatory reactions in all nano-extract treated groups and prevented it completely in nano-extract post-treated group. Moreover, it prevented the qualitative alterations in the different native patterns and isoenzymes in nano-extract post-treated group through hiding abnormal bands with restoring normal ones. Also, it prevented the quantitative alterations in all nano-extract treated groups and restoring the relative quantities of the total native bands to control values. As regard to the molecular assay, nano-extract decreased expression of the tumor suppressor genes (*TP*^53^ and *APC*) in all nano-extract treated groups but it restored their expression levels to normal values in nano-extract post-treated group.
